# In Vitro and In Vivo Testing to Determine Cd Bioaccessibility and Bioavailability in Contaminated Rice in Relation to Mouse Chow

**DOI:** 10.3390/ijerph16050871

**Published:** 2019-03-10

**Authors:** Shuo Sun, Xiaofang Zhou, Zhian Li, Ping Zhuang

**Affiliations:** 1 South China Botanical Garden, Chinese Academy of Sciences, Guangzhou 510650, China; sunshuo16@scbg.ac.cn (S.S.); zxfang2017@scbg.ac.cn (X.Z.); 2 College of Resources and Environment, University of Chinese Academy of Sciences, Beijing 100049, China

**Keywords:** cadmium, bioaccessibility, bioavailability, absorption, mineral elements, liver, kidney, contaminated rice

## Abstract

A combination of an in vitro physiologically based extraction test (PBET) and an in vivo mouse model was used to determine Cd oral bioaccessibility and estimate bioavailability in Cd-contaminated rice. The PBET found lower Cd bioaccessibility in the intestinal stage (40–50%) than in the gastric stage (93–98%) for both rice and mouse chow. No significant difference was found in Cd bioaccessibility between contaminated rice and Cd-amended mouse chow in the gastric or gastrointestinal phase (except for rice 1). The result of the in vivo bioassay revealed that Cd absorption in the kidney or liver of mice fed with contaminated rice were significantly higher than in the mouse chow group containing an equal Cd concentration. Correlation analysis between concentrations of different elements in mouse chow or rice and Cd concentrations in mice kidney or liver showed that Fe, Ca, Cu, and Zn had significant negative correlation (r^2^ > 0.7, *p* < 0.01). These results suggest that nutritional elements in the diet could affect Cd absorption and distribution in organs and that different food matrices may result in unequal Cd health risks at an equal Cd concentration due to the specific mineral content of food.

## 1. Introduction

Cadmium (Cd), one of the heavy metals, is known to accumulate in plants and animals from several pathways of environmental exposure. In animals, Cd exposure is associated with the etiology of cardiovascular disease, renal dysfunction, and bone disease, such as osteoporosis [1,2]. Rice intake may be a major contributor to Cd exposure because rice is a staple food for half of the world’s population and is the cereal crop that accumulates Cd in the highest concentration [3,4]. An extensive nationwide survey showed that 65% of all field rice produced around mine-impacted areas in China exceeded national standards for the permittable limit of Cd in food [5]. Thus, rice safety has been considered as a critical issue, and there is an urgent need to assess health risks from ingesting Cd-contaminated rice.

As with all minerals, the ingested dose of Cd does not reflect the actual level of Cd that is absorbed by the human body [6]. A greater understanding of the bioaccessibility and bioavailability of Cd is required to assess its risks to human health [7,8]. Oral bioaccessibility (BAC) is theoretically the amount of substance that is soluble in the gastrointestinal environment, i.e., the fraction that is released from the matrix into the gastrointestinal tract during the digestion process and, thus, becomes available for intestinal absorption and can enter the bloodstream [9]. Several in vitro approaches have been developed in attempts to mimic the effects of the digestion process in mammals [10]. The physiologically based extraction test (PBET), an in vitro test system for predicting the bioavailability of metals from a solid matrix, is the most widely used to date [11] and was previously used to determine bioaccessible portions of both essential and toxic elements in contaminated soils or some food matrix samples, such as raw vegetables [12], uncooked [13] and cooked rice [5,14] and seafood [15].

To accurately evaluate and characterize the health effects and health risks of Cd in food or soils, many researchers have used in vivo mice, rat, or swine bioassays to determine the bioavailability (the fraction of the externally administered amount of a metal substance that is absorbed and reaches the systemic circulation or central compartment of the receptor) of Cd in endpoints (e.g., liver and kidney) [6,16,17]. The bioaccessibility encompasses what is actually available plus what is potentially available physiologically. A steady-state model with Cd accumulation in kidney, liver, or femur as the endpoints was used with a mouse bioassay by daily administration of Cd-contaminated soil over 10 days [16]. For metal bioavailability, most studies have focused on arsenic (As), Cd, and lead (Pb) in contaminated soil [18,19]. A limited number of studies have addressed Cd bioavailability in food matrices [7,20,21]. There is a lack of studies on the bioavailability of Cd in different food matrices containing equal concentrations of Cd. 

Several studies have suggested that the bioavailability of metal is different for different food sources and numerous factors influence intestinal absorption and organ retention of Cd, such as mineral status [21,22]. For example, Reeves and Chaney [7] reported in rats that Zn, Fe, and Ca modulate the absorption and organ retention of Cd from sunflower kernels. Since rice safety is an issue of increasing concern, there may be differences in Cd gastrointestinal absorption and organ retention of Cd in mice fed with contaminated rice versus Cd-amended mouse chow. Such a study may reveal potential interactions between Cd and other nutritional elements. Investigating the bioaccessibility and bioavailability of Cd in mice fed Cd-contaminated rice would help to establish an accurate risk assessment method aimed at decreasing Cd absorption. 

Therefore, the aims of this study were to (1) compare the potential bioaccessibility based on an in vitro extraction test and real bioavailability of Cd using a mouse model in the contaminated rice and Cd-amended mouse chow, and (2) determine whether the nutritional status of different food matrices affect Cd accumulation and absorption. This work is a preliminary step in the health risk assessment of Cd from different food sources, and can also guide future risk reduction of Cd from dietary strategies.

## 2. Materials and Methods

### 2.1. Chow and Rice Preparation

Two rice samples were collected from farmers’ markets located in Shaoguan, Guangdong Province, China. This area is contaminated by the wastewater and discards of mining operations. Mouse chow (1 kg) power was mixed with 1 or 4 mg standard Cd chloride solution and equilibrated for 3 days to achieve concentrations of 0.99 and 3.64 mg Cd kg^−1^, dry weight (dw). These concentrations were chosen as they represent Cd concentrations equivalent to the selected rice in this study. Rice was cooked with deionized water at a water to rice ratio of 2:1 (*v*/*v*). Cooked rice and Cd-amended mouse chow were molded into pellets and freeze-dried to constant weight. 

### 2.2. In Vitro Bioaccessibility Test

The bioaccessibility of Cd in mouse chow and rice was determined by the PBET model [9]. This in vitro extraction procedure makes use of simulated gastric and intestinal juices that are applied to samples to try to predict the availability of Cd for human absorption. According to the method, 1 L simulated gastric solution contained 1.25 g pepsin, 0.5 g sodium malate, 0.5 g sodium citrate, 420 µL lactic acid, and 500 µL glacial acetic acid and the pH was adjusted to 1.5 ± 0.1 with HCl [11]. Then, 0.3 g of Cd-amended mouse chow or cooked-dried rice was mixed in a 50 mL centrifuge tube with 30 mL of simulated gastric juice. The mixture was incubated in a thermostatic bath at 37 °C for 1 h at 150 rpm. Then, the solution was centrifuged at 4000 rpm for 15 min, and a 5 mL aliquot was collected and filtered through a 0.45 µm filter membrane for analysis. For the simulated gastrointestinal stage, 52.5 mg of bile salt and 15 mg of pancreatin were added to each tube and the pH was adjusted to 7.0 with saturated NaHCO_3_ [11]. The samples were placed in a thermostatic bath at 37 °C and incubated at 150 rpm for 2 h and then centrifuged. The extracts from the gastric and intestinal phases were kept at 4 °C and then measured as soon as possible. 

### 2.3. In Vivo Experiment of Cd Exposure

Animal work was approved by the Animal Ethics Committee. Female Balb/C mice (about 6 weeks) were used in the in vivo study to determine Cd bioavailability. Mice were acclimated for 7 days in metabolic cages (temperature of 22 ± 2 °C, 12/12 h light/dark cycle) where they were given commercial mouse chow and allowed to drink tap water ad libitum. Mice were then randomly assigned to cages with three mice per cage. Following fasting overnight, mice were weighed and fed with Cd-amended mouse chow 1 (0.99 mg Cd kg^−1^, dw), chow 2 (3.64 mg Cd kg^−1^, dw), Cd-contaminated rice 1 (1.26 mg Cd kg^−1^, dw), and rice 2 (3.65 mg Cd kg^−1^, dw) for 10 days and allowed to drink tap water ad libitum. Mice fed with basal mouse chow were used as a control group. All treatments were replicated three times. Food supplied and remaining were recorded accurately every day. At the end of the 10-day period, mice were fasted overnight, weighed, and sacrificed to collect kidney, liver, and femur samples, which were stored in a plastic bag. Femur samples were collected to examine potential Cd effects on bone diseases. All wet samples were immediately stored at −80 °C and then freeze-dried and stored in a moisture-proof plastic bag prior to digestion. 

### 2.4. Element Analysis

All chemicals were of analytical reagent grade and purchased from Sigma. Cooked-dried rice powder, mouse chow powder, and organ samples were digested with nitric acid (Sigma company, St. Louis, MO, USA) using a microwave digestion instrument (Multiwave PRO, Anton Paar, Graz, Austria) and diluted with ultrapure water. The concentrations of Cd and nutritional elements in the mouse chow, rice, and the extracts were determined by graphite furnace atomic absorption spectrometry (GFAAS, analytikJena novAA800, Jena, Germany) or flame atomic absorption spectrometry (FAAS, analytikJena novAA800, Jena, Germany). To ensure the quality of the analytical procedures, a national standard material (rice GBW(E) 10010 and pork liver GBW 10051) and blanks were included in the digestion batches. The recoveries of Cd, Fe, Ca, Zn, Mg, and Cu in the reference material are presented in Table S1. The calibration curve for Cd measurement was prepared using the standard GNM-M27195-2013 solution. The evaluation of the obtained data was conducted using a standard addition method. 

### 2.5. Statistical Analysis

The bioaccessibility (%) of Cd was calculated by dividing the extractable Cd in the gastric or gastrointestinal phase of the in vitro method by total Cd in the rice or mouse chow [9]:

(1) $$\mathrm{Bioaccessibility}\;(\%)\;=\;\frac{\mathrm{extractable}\;\mathrm{Cd}}{\mathrm{total}\;\mathrm{Cd}}\;\times \;100$$

Absorption of Cd that accumulated in mouse organs was calculated using the Cd concentration (micrograms per kilogram of fresh weight) together with the weight coefficient of individual mouse organs, according to the following formula [17]:

(2) $$\mathrm{Absorption}\;(\mu g)\;=\;C\;\times \;\mathrm{mouse}\;\mathrm{weight}\;(\mathrm{kg})\;\times \;\frac{\mathrm{organ}\;\mathrm{weight}}{\mathrm{mouse}\;\mathrm{body}\;\mathrm{weight}}\;\times \;10^{-2}$$

where absorption is the total amount of Cd in the organ (µg), and C is the Cd concentration in the organ (µg kg^−1^ FW).

The absorption efficiencies of Cd in various tissues was calculated as follows:

(3) $$\mathrm{Absorption}\;\mathrm{efficiency}\;(\%)\;=\;\frac{\mathrm{absorption}}{\mathrm{dose}}\;\times \;100$$

where dose is the Cd dose level from the rice or amended mouse chow.

All data analyses were performed using SPSS software (2018 SPSS Inc., Chicago, IL, USA) and Excel 2016 (Copyright Microsoft Excel 2016, Redmond, WA, USA) and presented as the mean and standard deviation. In order to compare the differences in organ Cd absorption and the comprehensive effects after exposure between mice fed rice and mice fed chow, the data of the “rice group” and “chow group” in Figure 2 were combined. One-way ANOVA based on the least significant difference (LSD) or Turkey HSD was applied to determine the difference between the rice group and the mouse chow group. 

## 3. Results and Discussion

### 3.1. Rice and Mouse Chow Characteristics

As shown in Table 1, the Cd concentrations in rice 1 and 2 were 1.26 and 3.65 mg kg^−1^, respectively, which exceeded the maximum allowable values of 0.4 and 0.2 mg kg^−1^ established by the Joint FAO/WHO Expert Committee on Food Additives [23] and China [24], respectively. These two types of rice containing elevated Cd were collected from farmers’ markets around mine-impacted areas [4]. Meanwhile, the mouse chow was amended with Cd chloride to obtain an equal Cd concentration of 0.99 mg kg^−1^ (chow 1) and 3.64 mg kg^−1^ (chow 2), whereas the background Cd level was 0.12 mg kg^−1^ in untreated mouse chow. The Ca, Mg, Fe, and Zn concentrations in the mouse chow were 11,300, 2700, 110, and 113 mg kg^−1^, which were 47–87, 2.1–2.7, 2.2–3.7, and 3.8–5.6 times higher than those in rice 1 and rice 2, respectively (Table 1). 

### 3.2. In Vitro Bioaccessibility of Cd

**Figure 1:**
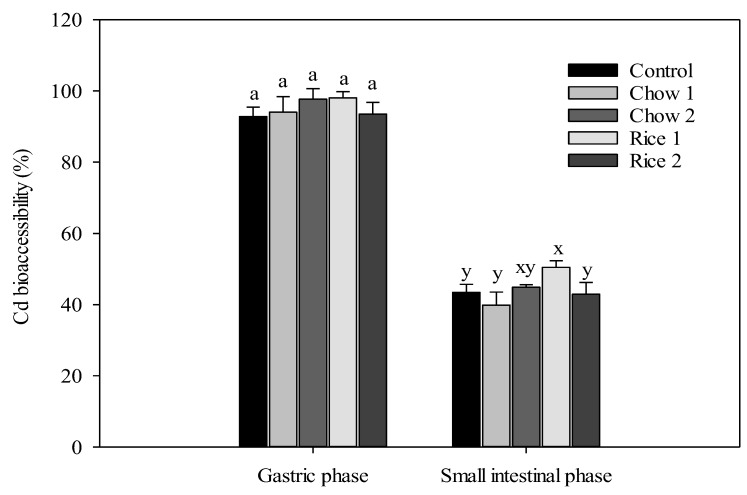
The bioaccessibility of cadmium (Cd) in the gastric phase and gastrointestinal phase in mouse chow amended with Cd chloride (Control: 0.1 mg kg^−1^; Chow 1: 0.99 mg kg^−1^; and Chow 2: 3.64 mg kg^−1^) or contaminated rice (Rice 1: 1.26 mg kg^−1^ and Rice 2: 3.65 Cd mg kg^−1^) (mean ± SD, *n* = 3). Different letters above the columns indicate statistical difference between different treatments (*p* < 0.05).

The bioaccessibilities of Cd in contaminated rice and mouse chow as determined by the in vitro PBET method are presented in Figure 1. In the gastric phase, the average Cd bioaccessibilities were 93%, 94%, and 98% for control chow, chow 1, and chow 2, respectively. The Cd bioaccessibilities in rice 1 and rice 2 were 98% and 94%, respectively. No significant difference was found in bioaccessible Cd concentration in gastrointestinal extracts when comparing mouse chow and rice. In the gastrointestinal phase, the average Cd bioaccessibilities were 43%, 40–45%, and 43–50% of that in control chow, Cd-amended mouse chow, and rice, respectively (Figure 1). In our previous studies, Zhuang et al. [25] reported similar percentages of Cd bioaccessibility in cooked contaminated rice of 86% in the gastric phase and 51% in the intestinal phase using the Unified Bioaccessibility Research Group of Europe (BARGE) in vitro method (UBM). The present results are in agreement with those of previous studies [5,26] showing that Cd bioaccessibility is typically ~50% lower in the gastrointestinal phase than in the gastric phase. The lower release of Cd in the gastrointestinal phase may be due to its higher pH versus that of the gastric fluid (pH 7.0 vs. 1.5, respectively). Under intestinal conditions, Cd coprecipitation with Fe or absorption onto Fe oxides via surface complexation of ligand exchange may occur, resulting in a decrease in Cd bioaccessibility [22]. 

When compared to Cd-amended chow 1, a significant increase in Cd bioaccessibility in the gastrointestinal fraction of rice 1 was observed. Previous studies suggested that nutritional elements including Ca, Fe, Mg, and Zn can affect Cd during its gastrointestinal transit [26]. For example, [27] reported that CaCl_2_ and FeCl_3_ can significantly reduce the bioaccessibility of Cd and Pb in some vegetables. Moreover, when assessing Cd bioaccessibility, a specific in vitro extraction test as well as mineral characteristics of the food matrix should be considered [28,29].

### 3.3. In Vivo Bioavailability of Cd

**Figure 2:**
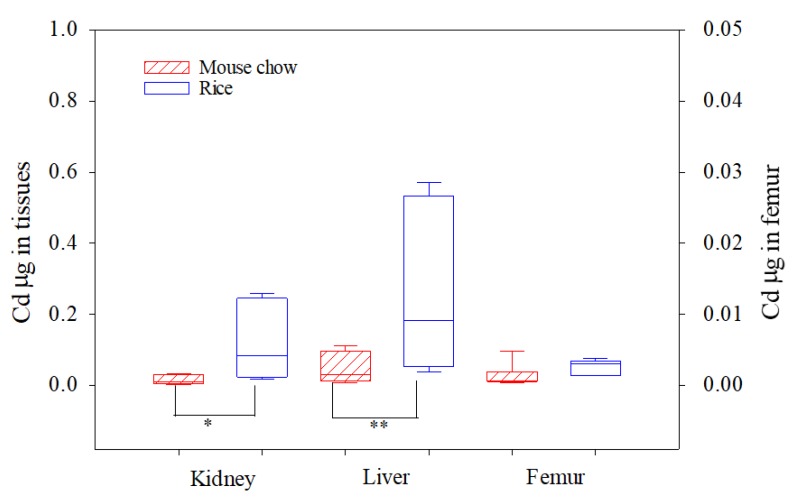
Absorption of Cd (µg) per mouse kidney, liver, and femur after 10 days of Cd exposure. Significant differences between chow and rice groups (* *p* < 0.05, ** *p* < 0.01) based on ANOVA and Tukey’s honestly significant difference (HSD) tests. Please note the different scale for the femur Cd values.

After 10 days of steady state dosing with Cd using an in vivo mouse model, the liver, kidneys, and femurs of mice were analyzed as biomarkers for Cd absorption determination. No significant differences were found in the mean food intake during the experimental period among the Cd-amended mouse chow group (2.76 g d^−1^), contaminated rice group (2.68 g d^−1^), and the control group (2.84 g d^−1^). The average weights of mice kidneys, liver and femurs in different treatment groups after 10 days of Cd exposure are presented in Table S2. In the present study, the in vivo bioavailability of Cd was evaluated in three ways: (1) bioavailable organ Cd concentrations (µg kg^−1^, Fw) (Table 2); (2) absorption of Cd (µg Cd per organ) (Figure 2), and (3) absorption efficiencies of Cd (%) in different organs (Table 3). 

Table 2 shows data on the Cd concentrations in kidney, liver, and femur based on µg Cd kg^−1^ fresh weight (fw). The background Cd levels in kidney, liver, and femur, as determined in the control group, were 4.9, 3.5, and 1.6 µg kg^−1^ fw, respectively, which were lower than those reported in the study of Schilderman et al. [16]. As shown in Table 2, the amounts of bioavailable Cd that accumulated in the liver and kidney increased significantly with increasing doses of Cd in the amended mouse chow and contaminated rice. In individual mice, good linear relationships (r^2^ = 0.92–0.97, *p* < 0.01) were found between the accumulation of Cd in the kidney or liver and Cd initial dose after 10 days exposure to either Cd-contaminated rice or Cd-supplemented mouse chow, illustrating the suitability of using these biomarkers to study the bioaccumulation of chronic levels of Cd in mammalian kidney and liver (Figure S1). There was a significant increase in the Cd concentration in the three tissues from the Cd-amended mouse chow or contaminated rice groups compared with the control group (*p* < 0.05). In kidneys from the Cd-amended chow groups, Cd concentrations of 23 µg kg^−1^ and 63 µg kg^−1^ for chow 1 (amended with 1 mg Cd kg^−1^) and chow 2 (4 mg Cd kg^−1^), respectively, were found, whereas in the contaminated rice group, 107 and 290 µg kg^−1^ was found for rice 1 (1.26 mg Cd kg^−1^) and rice 2 (3.65 mg Cd kg^−1^). A similar tendency was found in the results of the liver Cd accumulation, showing higher liver Cd concentration (84–219 µg kg^−1^) in the contaminated rice group than in the Cd-amended chow group (16–57 µg kg^−1^). Reeves and Chaney [30] reported Cd concentrations of 34 µg kg^−1^ and 45 µg kg^−1^ in the liver and kidney, respectively, of female rats that were fed a basal diet without added Cd. In the present study, the femurs did not accumulate high concentrations of Cd when mice were exposed to high doses of Cd, implying that the femur is not a good biomarker for measuring Cd bioavailability in contaminated rice based on a mouse model. This result is in line with the literature [16]. 

Daily food consumption by mice was about 2.68–2.76 g d^−1^ rice or chow pellets per day per mouse over the 10-day experimental period. Generally, mice fed with contaminated rice exhibited significantly higher Cd absorption in the kidney and liver than did the mouse chow group (Figure 2). With respect to both Cd-amended mouse chow and contaminated rice groups, the absorption detected in the liver (0.02–0.54 µg per organ) was higher than that in the kidney (0.01–0.25 µg per organ). This result is in accordance with previous studies indicating that after an oral dose of CdCl_2_, Cd mainly accumulates in the liver, followed by the kidneys [17]. No difference was found in femur Cd absorption among the treatment groups.

The absorption efficiencies of Cd in the kidney, liver, and femur are presented in Table 3. In the present study, after a 10-d feeding trial, 0.05% and 0.11% of the original total dose of Cd accumulated in the kidney and liver of the control group, whereas 0.02% and 0.05–0.06% of the initially administered dose was recovered in the same organs of the CdCl_2_-amended chow groups. In the rice fed groups, 0.08–0.09% and 0.18–0.2% of the initially administered Cd doses were stored in the kidney and liver, respectively. These data are lower than the findings of Reeves and Chaney [7] who found that the amount of ^109^Cd retained in the liver and kidney of female rats after 15.5 days exposure ranged from 0.15–1.5% and 0.15–1.1% of the initial dose, respectively. The present results agree with other studies showing a low bioavailability (0.12–0.22%) of Cd in nonlactating ewes [31]. These results are lower than those from previous investigations showing the absorption efficiencies in different species (e.g., rats) after an oral dose of CdCl_2_ varied from 0.5–8% [17,32].

It is worth noting the significantly (*p* < 0.05) increased tissue absorption efficiency of Cd in the rice-fed mice compared to the Cd-amended chow group. For example, the absorption efficiencies of Cd in the kidney and liver of the low-Cd rice (1.26 mg kg^−1^) group were 6.7 and 6.1 times higher than those of the mouse chow group containing a comparable Cd concentration (0.99 mg kg^−1^). A similar trend of Cd absorption efficiency was found between the high-Cd rice (3.65 mg kg^−1^) and high-Cd mouse chow (3.64 mg kg^−1^) groups. More Cd may enter the internal organs (kidney and liver) when dietary Ca, Mg, Fe, and Zn are marginal in rice versus the higher mineral content of mouse chow [26,30]. Groten et al. [33] and Walter et al. [34] also reported that dietary mineral supplements containing Fe, Ca, and Zn could significantly reduce the accumulation and toxicity of Cd in growing rats.

### 3.4. Correlation of Cd Accumulation in Biomarkers and Mineral Elements

To ascertain the relationship of bioavailable Cd concentration in biomarkers and essential dietary minerals, the Pearson correlation coefficient (r) was calculated to compare these variables (Table 4). High negative correlation was found between Fe and Cd (r = −0.92). Similarly, significant negative correlations between Ca and Cd (r = −0.80, *p* < 0.01), Mg and Cd (r = −0.68, *p* < 0.01), Cu and Cd (r = −0.81, *p* < 0.01), and Zn and Cd (r = −0.80, *p* < 0.01) were found, indicating that essential dietary minerals, such as Zn, Fe, and Ca might modulate the metabolism of Cd and affect the organ concentration and absorption of Cd. Kim et al. [35] reported that the uptake of Cd is associated with the Fe divalent metal transporter 1 (DMT1). Cadmium and Fe may compete for binding sites on DTM1, effectively inhibiting gastrointestinal absorption of Cd when the diet is rich in Fe. Similar to Fe, it has been suggested that the low-molecular-weight calcium-binding protein (CaBP) has a close relationship with the absorption of Cd because the binding affinity of CaBP for Cd and Ca is similar [36,37]. This study showed a higher kidney and liver Cd absorption efficiency in mice fed with a diet low in essential minerals (Table 3) versus mice fed adequate amounts of mineral elements (Table 2). Similarly, in rats fed rice diets, adequate intake of Fe and Ca combined caused a 6-fold decrease in Cd absorption compared to that in rats consuming the same marginal minerals [19]. The finding from Zhao et al. [38] also confirmed our result that increased dietary levels of Ca and Fe significantly decreased relative bioavailability of Cd in rice. Moreover, Europeans and Americans who consume a variety of garden foods containing higher Zn, Fe, and Ca along with Cd have lower Cd absorption [21]. Since food matrices differ in Ca, Fe, and Zn concentrations, diet appears to influence Cd absorption and accumulation suggesting that other minerals can modulate Cd toxicity [7,39].

### 3.5. Comparison of Cd Bioaccessibility vs Bioavailability and Implication for Exposure Assessment 

In vitro extraction tests and in vivo bioassays, known as oral bioaccessibility and bioavailability tests, respectively, are important approaches to assess the health risk of toxicants to humans [20]. The bioaccessible part of Cd evaluated in the simulated human gastrointestinal system may not reflect the actual fraction that is available for gastrointestinal metal absorption [40]. This was reflected by higher Cd bioaccessibility based on in vitro extraction test (Figure 1) compared to the absorption efficiency of Cd based on the in vivo bioassay (Table 3). Thus, in vivo bioavailability tests are likely more physiologically relevant and, where possible, should be employed in risk assessments of human exposure to Cd via rice consumption.

The mineral content of food can affect Cd accumulation and absorption [21]. We observed a similar confirmation in the present study, wherein mice fed with Cd-amended chow (0.99 or 3.64 mg Cd kg^−1^) containing adequate nutritional elements showed a significant decrease (around 4–5 folds) in Cd absorption and accumulation compared to mice fed contaminated rice (1.26 or 3.65 mg Cd kg^−1^) with low levels of Ca, Zn, and Fe. Since Cd-contaminated rice is sold for human consumption in some areas impacted by mining, this might be an alternative approach to inhibiting Cd absorption in humans [7]. Populations who are nutritionally adequate with respect to Ca, Zn, and Fe may be at a lower risk of Cd-related diseases than those who are nutritionally marginal when consuming Cd-contaminated rice.

## 4. Conclusions

Using an in vivo mouse model, we demonstrated that the bioavailability of Cd from rice in mice is different from that of commercial mouse chow. The results of our experiment indicated heterogeneity between potential bioaccessibility determined by the in vitro method and estimated bioavailability determined using an in vivo bioassay because large Cd bioaccessible fractions from food matrices did not lead to elevated Cd concentrations in mouse tissue. An important innovative aspect of this study is the application of a new method for absolute bioavailability evaluation based on the total element amount in specific organs or whole organ systems. Using the measure of absorption, we were able to detect differences in Cd accumulation between contaminated rice and Cd-amended chow groups more accurately. The data presented suggest that mammalian mineral nutrient status may play a major role in determining how much dietary Cd is absorbed and retained in the body.

## Figures and Tables

**Table 1:** Concentrations of major and minor elements in basal mouse chow and rice (mean ± SD, *n* = 3).

Constituent	Basal Mouse Chow	Rice 1	Rice 2
Major elements (mg kg^−1^)			
Ca	11300 ± 200	240 ± 10	130 ± 10
Mg	2700 ± 20	1000 ± 100	1270 ± 50
Fe	110 ± 20	50 ± 10	30 ± 10
Minor elements (mg kg^−1^)			
Cd	0.12 ± 0.01	1.26 ± 0.03	3.65 ± 0.05
Pb	0.13 ± 0.01	0.89 ± 0.04	0.05 ± 0.01
Cu	16.0 ± 0.19	6.03 ± 0.30	4.12 ± 0.15
Zn	113 ± 0.70	30.1 ± 0.40	20.2 ± 0.06

**Table 2:** Cadmium concentrations in mouse kidney, liver, and femur when mice were supplied with contaminated rice or Cd-amended mouse chow for 10 days (µg kg^−1^ FW, mean ± SD, *n* = 3).

Treatments	Kidney	Liver	Femur
Control	4.90 ± 3.1d	3.5 ± 0.3c	1.6 ± 0.2a
Chow 1	22.5 ± 0.8d	15.7 ± 0.6c	1.0 ± 0.1a
Chow 2	62.8 ± 2.5c	57.3 ± 4.1b	4.7 ± 1.9a
Rice 1	106.8 ± 6.1b	84.4 ± 13.7b	2.9 ± 0.3a
Rice 2	290.7 ± 11.9a	218.5 ± 12.3a	3.1 ± 0.2a

Mouse chow was amended with Cd chloride: Control: 0.1 mg Cd kg^−1^; Chow 1: 0.99 mg Cd kg^−1^; and Chow 2: 3.64 mg Cd kg^−1^. Contaminated rice purchased from farmers’ markets: Rice 1: 1.26 mg kg^−1^; Rice 2: 3.65 Cd mg kg^−1^. Values with different letters within the same organ indicate significant difference at p < 0.01 based on the least significant difference (LSD) test.

**Table 3:** The absorption efficiencies (%) of Cd in mouse organs based on the Cd concentrations in mouse kidney, liver, and femur after 10 days of Cd exposure.

Treatments	Kidney (%)	Liver (%)	Femur (%)
Control	0.050 ± 0.020 ^b^	0.110 ± 0.001 ^b^	0.0090 ± 0.0010 ^a^
chow 1	0.020 ± 0.002 ^c^	0.050 ± 0.005 ^c^	0.0010 ± 0.0001 ^c^
chow 2	0.020 ± 0.001 ^c^	0.060 ± 0.004 ^c^	0.0020 ± 0.0010 ^bc^
rice 1	0.090 ± 0.010^ a^	0.200 ± 0.040 ^a^	0.0030 ± 0.0010 ^b^
rice 2	0.080 ± 0.004^ a^	0.180 ± 0.007 ^a^	0.0010 ± 0.0002 ^c^

Mouse chow was amended with Cd chloride: Control: 0.1 mg Cd kg^−1^; Chow 1: 0.99 mg Cd kg^−1^; and Chow 2: 3.64 mg Cd kg^−1^. Contaminated rice purchased from farmers’ markets: Rice 1: 1.26 mg kg^−1^ and Rice 2: 3.65 Cd mg kg^−1^. Values with different letters (a, b, c) within the same organ indicate significant difference at *p* < 0.01 based on the least significant difference (LSD) test.

**Table 4:** The correlation between Cd concentration in mouse organs and the concentration of major and minor elements in mouse chow and contaminated rice.

Elements	Kidney	Liver	Femur
Ca	−0.799 **	−0.790 **	−0.062
Fe	−0.921 **	−0.921 **	−0.266
Mg	−0.679 *	−0.665 *	−0.008
Cu	−0.814 **	−0.798 **	−0.014
Zn	−0.797 **	−0.781 **	−0.025
Pb	−0.218	−0.218	−0.030

* indicates a significant correlation at the level of 0.05, ** indicates a significant correlation at the level of 0.01.

## Supplementary Materials

The following are available online at https://www.mdpi.com/1660-4601/16/5/871/s1, Figure S1: Relationship between Cd accumulation in mouse organs and exposure dose when mice were supplied Cd-amended mouse chow (A) or contaminated rice (B) for 10 days. Mouse chow was amended with Cd chloride, Control, 0.1 mg Cd kg^−1^; Chow 1, 0.99 mg Cd kg^−1^ and Chow 2, 3.64 mg Cd kg^−1^. Contaminated rice purchased from farmer’s market, Rice 1, 1.26 mg kg^−1^ and Rice 2, 3.65 Cd mg kg^−1^. Please note the different scale of femur Cd values, Table S1: Analytical data quality assessment (mg kg^−1^, mean ± SD, *n* = 3), Table S2: The average weights of mice kidney, liver and femur in different treatment groups after 10-day of Cd exposure (g, mean ± SD, *n* = 3).
