# Supplementary material for: In Vitro and In Vivo Testing to Determine Cd Bioaccessibility and Bioavailability in Contaminated Rice in Relation to Mouse Chow
Source: Int J Environ Res Public Health. 2019 Mar 10;16(5):871. doi: 10.3390/ijerph16050871 (PMC6427773; doi:10.3390/ijerph16050871)
Supplement: Supplementary file 1 [file ijerph-16-00871-s001.pdf]

Supplementary

In Vitro and In Vivo Testing to Determine Cd Bioaccessibility and Bioavailability in Contaminated Rice in Relation to Mouse Chow

Shuo Sun, Xiaofang Zhou, Zhian Li and Ping Zhuang

Table S1. Analytical data quality assessment (mg kg^{-1}, mean ± SD, n = 3).

Metals	Certified value	Determined value
	CRM-GBW10051 (pork liver)	
Cd	1.0 ± 0.07	0.99 ± 0.05
Cu	52 ± 3	40.9 ± 1.6
Zn	211 ± 11	171 ± 7
Mg	630 ± 40	622 ± 3
Fe	519 ± 34	488 ± 9
Ca	230 ± 30	196 ± 2
	CRM-GBW10010 (rice)	
Cd	0.087 ± 0.005	0.080 ± 0.002
Cu	4.9 ± 0.3	3.90 ± 0.13
Zn	23 ± 2	17.5 ± 0.6
Mg	410 ± 60	379 ± 11
Fe	7.6 ± 1.9	6.7 ± 0.1
Ca	110 ± 10	93 ± 7

Table S2. The average weights of mice kidney, liver and femur in different treatment groups after 10-day of Cd exposure (g, mean ± SD, n = 3).

	kidney	liver	femur
Control	0.75 ± 0.30	2.04 ± 0.30	0.38 ± 0.04
Chow 1	0.68 ± 0.20	1.80 ± 0.03	0.48 ± 0.02
Chow 2	0.49 ± 0.01	1.77 ± 0.04	0.64 ± 0.11
Rice 1	0.80 ± 0.01	2.41 ± 0.20	1.03 ± 0.04
Rice 2	0.85 ± 0.02	2.51 ± 0.10	1.08 ± 0.03

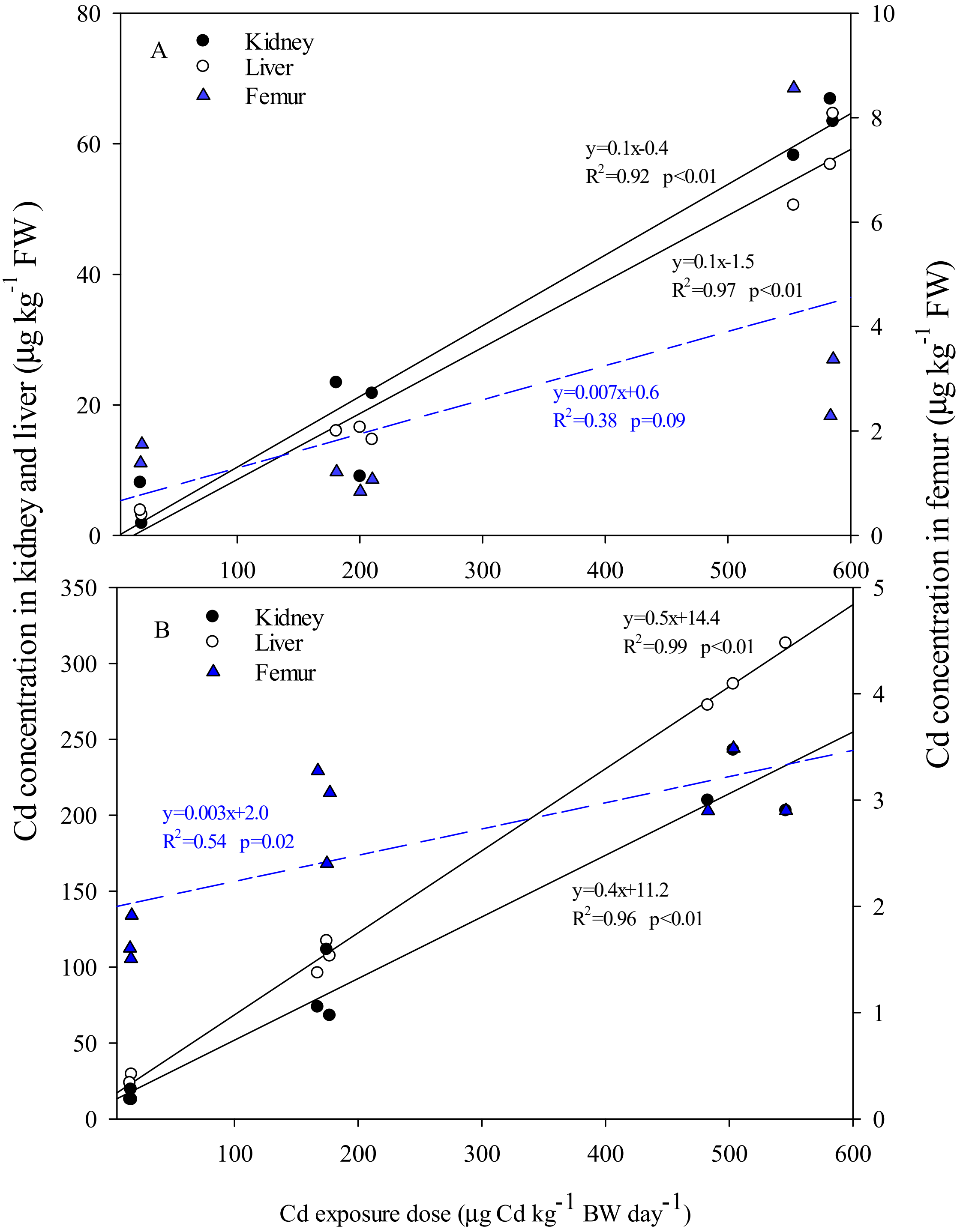

Figure S1. Relationship between Cd accumulation in mouse organs and exposure dose when mice were supplied Cd-amended mouse chow (**A**) or contaminated rice (**B**) for 10 days. Mouse chow was amended with Cd chloride, Control, 0.1 mg Cd kg^{-1}; Chow 1, 0.99 mg Cd kg^{-1} and Chow 2, 3.64 mg Cd kg^{-1}. Contaminated rice purchased from farmer's market, Rice 1, 1.26 mg kg^{-1} and Rice 2, 3.65 Cd mg kg^{-1}. Please note the different scale of femur Cd values.
